# Engaging social media users with corporate social responsibility messages: An integrated theoretical approach in the automotive industry during social media era

**DOI:** 10.1371/journal.pone.0322481

**Published:** 2025-06-05

**Authors:** He Dong, Pan Zhao, Cindy Sing Bik Ngai

**Affiliations:** Department of Chinese and Bilingual Studies, The Hong Kong Polytechnic University, Hung Hom, Hong Kong; Czestochowa University of Technology: Politechnika Czestochowska, POLAND

## Abstract

Corporate Social Responsibility (CSR) messaging on social media has become a key strategy for companies to engage the public. However, limited research has investigated how specific CSR communication strategies influence public online engagement, particularly in the Chinese context. This study analyzed the effects of three dependent variables: (1) CSR message themes (aligned with ISO 26000 standards), (2) relationship cultivation strategies (Openness, Information Dissemination, Interactivity and Involvement, and Sharing Tasks), and (3) multimodal message types (text with emoticons, picutures, and videos) on social media users’ engagement behaviors (likes, shares, and comments). A quantitative content analysis was conducted on 421 CSR-related posts from the Weibo accounts of the top ten automotive brands in China, using Negative Binomial Regression (NB2) to examine the associations between CSR factors and user engagement. The findings revealed that consumer-related CSR themes significantly increased likes, shares, and comments. Relationship cultivation strategies, particularly Interactivity and Involvement, positively impacted engagement, especially in posts that encouraged commenting or sharing. Additionally, posts incorporating text with emoticons were found to significantly boost comments. Theoretically, this study integrates Stimuli-Response Theory’s focus on external stimuli with Uses and Gratifications Theory’s emphasis on users’ active motivations, offering a more comprehensive framework for understanding user engagement. Practically, it provides clear, actionable recommendations for automotive companies: prioritize consumer-focused CSR topics, adopt interactive strategies, simplify technical content, and utilize.

## 1. Introduction

CSR communication has become a key strategy for businesses to achieve sustained growth, strengthen stakeholder relationships, and gain competitive advantages [1]. Effective CSR communication enhances brand reputation [2], expands market share [3], and fosters customer loyalty [4]. Public engagement is a vital indicator of successful CSR communication, enabling corporations to gauge public reactions, understand stakeholder expectations, and build meaningful relationships [5,6].

The rise of social media has shifted CSR communication from traditional channels—such as newspapers and corporate websites—to interactive, multimodal online platforms. Social media facilitates two-way communication, empowering corporations to directly engage stakeholders [7]. Its multimodal capabilities allow for diverse content types, including images, videos, and emoticons, which enhance public engagement [8–10].

Despite extensive research on CSR across marketing, management, and communication disciplines [11], several critical gaps remain. First, studies often focus on either corporate CSR strategies or public engagement behaviors (e.g., likes, shares, comments), with limited attention to how corporate messaging influences engagement in social media context. Second, most research examines textual content, overlooking the growing use of multimodal elements to boost public interaction. Third, CSR communication in controversial industries, such as the automotive sector, remains underexplored, particularly in the Chinese social media environment. Given China’s global leadership in automotive sales for twelve consecutive years [12], understanding how Chinese automotive companies use social media for CSR communication is both timely and critical.

This study addresses these gaps by investigating how CSR communication strategies employed by leading Chinese automotive companies influence user engagement on Sina Weibo, China’s largest social media platform. Specifically, it examines three core dimensions: CSR message themes (ISO 26000 framework), Rrelationship Cultivation Strategies (Openness, Information Dissemination, Sharing Tasks, and Interactivity/Involvement), and multimodal message types (text combined with pictures, videos, or emoticons). The study evaluates their impacts on user engagement: likes, shares, and comments.

Theoretically, this research integrates the Stimulus-Response theory’s focus on external stimuli with the Uses and Gratifications theory’ s emphasis on users’ active motivations, providing a comprehensive framework for understanding user engagement. By analyzing CSR themes, relationship strategies, and multimodal communication, it contributes to the literature by clarifying the interplay and combined effectiveness of these factors in driving engagement. Practically, the findings offer evidence-based guidance for automotive companies to design impactful CSR strategies on social media, enhancing corporate reputation, strengthening consumer relationships, and fostering meaningful stakeholder engagement in the digital age.

## 2. Literature review

### 2.1. CSR communication in the automotive industry

Controversial industries, such as tobacco, nuclear, and alcohol, face heightened public scrutiny due to the significant health, environmental, and social risks associated with their operations [13]. Their business practices often conflict with societal norms, making CSR communication both essential and challenging [14]. These challenges have driven growing scholarly interest in identifying effective CSR communication strategies for controversial industries [15].

The automotive industry exemplifies a controversial sector, marked by environmental pollution, heavy reliance on non-renewable resources, and occasional product-quality scandals. Consequently, automotive companies face mounting pressure from public to actively communicate their CSR efforts. Social media has become a key platform for such communication, allowing companies to disseminate CSR information, address public concerns, and foster relationships. According to a 2023 CAAM report, 98% of the world’s top 100 automotive brands actively communicate CSR initiatives on social media platforms like Facebook, Twitter, and Instagram, attracting significant public engagement.

Given the interactive nature of social media, scholars increasingly apply communication and psychological theories to understand online CSR engagement. S-R theory explains how specific communicative stimuli, such as CSR themes, elicit predictable audience responses [16]. U&G theory complements this by examining audience motivations for engaging with CSR content, such as seeking information, emotional connection, or social interaction. Additionally, Stakeholder theory [17] highlights the importance of targeted CSR messaging for managing diverse stakeholder relationships, while Legitimacy theory [18] highlights CSR communication as a means of maintaining societal acceptance.

Empirical research into automotive CSR communication has primarily focused on environmental, economic, and ethical dimensions. For instance, Shinkle and Spencer [19] found that environmental themes dominate global automotive CSR reports due to strong stakeholder expectations. Conversely, Ngai and Singh [6] observed that community involvement and development (CID) is a more prominent CSR theme among Chinese automotive firms than environmental concerns. These studies, despite their diffrent findings, demonstrate positive relationships between effective CSR themes and improved corporate reputation and performance.

However, existing research on CSR themes in the automotive sector remains limited, especially as companies increasingly address a broader range of CSR issues. Beyond traditional environmental, ethical, and economic concerns, automotive firms now communicate themes such as labor practices, human rights, fair operating practices, consumer issues and beyond. ISO 26000 offers a practical framework for categorizing these diverse CSR themes, enabling clearer and more consistent stakeholder communication. From an S-R theory perspective, ISO 26000 CSR themes can be viewed as strategic communication stimuli designed to elicit positive stakeholder responses, such as increased engagement. Given the expanding scope and complexity of CSR themes—and their critical role in stakeholder engagement—it is essential to investigate how automotive companies strategically use ISO 26000 themes to influence public responses. This research gap highlights the need for furtherly explore how CSR communication in the automotive sector drives engagement and fosters meaningful stakeholder relationships.

### 2.2 CSR Communication on social media

Social media is considered an ideal platform for corporations to disseminate CSR messages and build relationships with the public [20] due to its two key features—informative and interactive—that influence public engagement with CSR communication [21].

An informative strategy focuses on delivering accurate and coherent CSR messages, often utilizing multimodal content such as videos, images, and audio. These visual elements play a critical role in shaping public attitudes and behaviors by evoking emotions [8,22] and capturing the attention of users who quickly scroll through vast amounts of content [10]. Studies show that incorporating photographs in social media posts significantly increases likes, shares, and comments [23,24]. However, research has yet to determine which specific multimodal elements are most effective for driving public engagement in CSR communication, particularly in controversial industries.

An interactive strategy emphasizes engaging the public in the development and implementation of CSR initiatives. Research highlights how organizations use social media to foster two-way communication with stakeholders. For example, Rybalko and Seltzer [25] examined how Fortune 500 companies engage stakeholders on Twitter through dialogic communication, while studies on nonprofit organizations [26,27] explored strategies for fostering stakeholder engagement and dialogue. Despite these findings, limited research has examined how the public responds to interactive strategies embedded in CSR initiatives, leaving gaps in understanding their effectiveness.

Given these insights, it is essential to investigate how the automotive industry uses social media to cultivate relationships and enhance public engagement in CSR communication. Exploring how interactive and informative capabilities are employed can provide valuable guidance for fostering meaningful stakeholder relationships and maximizing engagement.

### 2.3 Relationship cultivation strategy in CSR communication

Given the importance of interactivity in social media for CSR communication, scholars have increasingly explored how corporations engage with the public to build healthy relationships. Relationship cultivation strategies, defined by Ki and Hon [28] as interactive behaviors aimed at fostering and maintaining effective relationships, have gained significant attention. These strategies are linked to positive outcomes such as mutual trust, recognition, satisfaction, and assistance—key indicators of effective communication between corporations and their audiences.

Previous studies have categorized relationship cultivation strategies into various groups. Stafford and Canary [29] identified five key strategies: Openness, Positivity, Networking, Assurances, and Sharing Tasks. With the rise of social media, three essential strategies for online relationship cultivation have emerged:

**Openness** refers to an organization’s willingness to engage in open and direct communication with the public. A social media platform makes it easy to ***share organizational information*** with its users. As Waters et al. [27] indicated, organizations should offer a comprehensive ***description*** of the organization, its history, goals and missions, use **hyperlinks** to lead users to its website, and use ***logos or other visual cues*** for intuitive identification [30].

**Disseminating Information** addresses the public’s concerns, needs and interests [30]. According to Kent and Taylor’s [31] argument, such ***disseminated information*** empowers the public to get involved in the organization as informed stakeholders. Corporations can use social media to ***make announcements***, to promote their products and to post photographs and videos. Additionally, ***hyperlinks*** can direct users to external content, such as media coverage and websites [30].

Relationships are cultivated through **Interaction and Involvement** [30]. McMillan et al. [32] categorized internet interaction as three types, namely “computer to human interaction” (e.g., ***navigation***), “human to human interaction” (e.g., ***make an advice to others, opportunities to contact the organization*** and ***share the information of their own page*** on social media) and “human to content interaction” (the ability to ***comment organizational messages*** and ***reply to others’ messages***) [30].

Researchers have examined how corporations employ social media to build online relationships. Chen and Rahman [33] found that Fortune 500 companies frequently used corporate blogs to foster customer relationships and a sense of community. Sharing Tasks and Networking were the most commonly employed strategies, with variations across industries.

However, most studies have focused on non-controversial industries, with limited research on sectors like the automotive industry. These industries face unique challenges, including enhancing credibility, sustaining stakeholder relationships, and addressing skepticism related to environmental and safety concerns. Furthermore, few studies have applied theories like S-R or U&G to explore how these strategies influence user engagement and strengthen relationships in CSR communication [30].

### 2.4 Multimodality in CSR communication

Social media’s informativity enables the use of multiple formats—videos, audio, visuals, and hyperlinks—in a single message. These multimodal elements can evoke emotions, influencing public engagement and behavior [8]. Multimodality has been shown to create meaning by emphasizing social actors, conveying authenticity, and establishing credibility [34]. It also transmits messages that words alone cannot fully convey [35].

Despite its potential, limited research has explored the role of multimodality in CSR communication, particularly non-verbal elements. While visuals are frequently used in CSR reports, corporate websites, and social media, their role in generating meaning remains underexplored [35]. Effective CSR communication requires analyzing both verbal and visual elements to understand how meaning is multimodally constructed [35].

In the automotive industry, research on multimodal CSR communication is scarce, with most studies focusing on advertisements [36]. Although multimodality has been studied in fields like education, medicine, politics, and AI, its application to CSR communication in this sector remains limited. Thus, a study examining the multimodal features of automotive companies’ CSR communications on social media and their impact on public engagement is urgently needed.

### 2.5 The engagement of Chinese Social Media Users in CSR Communication

Social media engagement is often measured by user interactions such as likes, shares, and comments [6,37], reflecting users’ interest and interaction with content from individuals or brands. In this context, CSR communication plays a key role in driving engagement by meeting societal expectations and responding to public opinion [6]. To foster participation and interaction, companies increasingly share CSR information on social media, employing its immediacy [6].

Research shows that engagement levels vary depending on CSR content [33,38]. For example, charitable donations tend to elicit stronger reactions than CSR efforts focused on ethical or environmental issues. However, there is limited understanding of how different types of CSR content influence engagement metrics and how other factors affect user interactions, particularly on Chinese social media platforms.

Despite China’ s economic growth and increased corporate adoption of CSR practices, research on CSR communication via Chinese platforms like Sina Weibo remains scarce [39,40]. As a leading platform, Weibo offers unique opportunities for firms to share CSR initiatives and engage users.

This study aims to analyze automotive corporations CSR posts on Weibo, the relationship cultivation strategies used, and the multimodal elements incorporated. It will also examine how these factors influence public engagement, measured by shares, likes, and comments.

## 3. Research questions

CSR themes in ISO 26000 define social responsibility, guide organizations in applying its principles, and promote best practices globally [41]. However, the CSR themes shared in messages and their impact on public engagement remain underexplored. Therefore, the first set of research questions is as follows:

RQ 1a. What were the CSR themes communicated by the leading automotive companies on Weibo?

RQ 1b Is there any association between each CSR theme and social media users’ engagement on Weibo in the form of shares, likes and comments?

Unlike traditional mass media, social media enables companies to engage and build relationships with users [7]. Relationship Cultivation Strategies are commonly used in CSR communication as they can enhance public engagement. However, studies on these strategies in car companies’ CSR messages and their link to public engagement remain limited. Therefore, the second set of research questions is as follows:

RQ 2a What strategies did automotive companies use on Weibo to cultivate relationships with social media users?

RQ 2b Is there any association between relationship cultivation strategies and public engagement on Weibo in the form of shares, likes and comments?

RQ 2c Is there any association between the subdimensions of each relationship cultivation strategy and public engagement on Weibo in the form of shares, likes and comments?

Social media’s informative feature allows for the extensive use of multimodal elements, such as pictures, videos, and emoticons, in CSR messages. Höllerer et al. [42] showed that multimodality enables complex and holistic communication, accommodating complexity and contradictions within a single issue. Despite its importance and widespread use, studies on multimodal elements in CSR messages remain scarce. Therefore, the third set of research questions is as follows:

RQ 3a What multimodal types were used in the CSR messages of automotive companies on Weibo?

RQ 3b Is there any association between each multimodal type and public engagement on Weibo in the form of likes, shares and comments?

## 4. Methodology

### 4.1. Theoretical framework

This study employs an integrated theoretical framework combining Stimulus-Response (S-R) theory [16] and Uses and Gratifications (U&G) theory [43] to analyze how automotive companies’ CSR communication influences users’ online engagement. S-R theory posits that external stimuli elicit predictable user responses, which, in this context, refers to CSR messages designed to trigger engagement behaviors such as likes, comments and shares [44]. U&G theory further explains why users engage with content, emphasizing their active role in seeking information, emotional satisfaction, or social interaction [45]. Combining these theories enables a deeper understanding of not only how CSR stimuli drive responses but also why specific strategies resonate with stakeholders, ultimately fostering outcomes like trust, reputation, and loyalty [45,46].

Using this framework, the study analyzes automotive companies’ CSR communication on social media across three dimensions: CSR themes, relationship cultivation strategies, and multimodal message characteristics. CSR themes, based on ISO 26000 guidelines, cover seven key areas, including human rights, labor practices, fair operating practices, environmental issues, consumer concerns, and community involvement [41]. These themes act as stimuli tailored to meet stakeholders’ informational, emotional, or social needs, thus prompting engagement.

Relationship cultivation strategies further enhance engagement. Drawing on Waters et al. [27] and Men and Tsai [30], these strategies include Openness, two-way communication, and collaborative actions, which encourage feedback, interaction, and public participation to build long-term relationships [45,46].

Finally, multimodal theory [47] highlights the role of text, images, videos, and emoticons in amplifying the impact of CSR communication on social media. Multimodal combinations, such as text paired with visuals or videos, effectively capture attention, enhance engagement, and improve corporate reputation [48].

By integrating these dimensions within the S-R framework, enriched by U&G theory, this study offers a comprehensive model to explain how CSR communication strategies engage stakeholders and drive positive outcomes.

### 4.2. Research design

This study utilized a quantitative content analysis approach [51] to address the research questions systematically. The analysis followed a multi-fold content analysis framework and employed the absence and presence coding method. For descriptive data related to RQs 1a, 2a, and 3a, SPSS was used to calculate the frequencies and percentages of CSR topics, relationship cultivation strategies, and multimodal types in the selected posts. Inter-rater reliability was assessed using Cohen’s kappa, ensuring the consistency and reliability of both the descriptive and subsequent statistical analyses.

The descriptive analysis identified the most prominent characteristics of CSR posts in terms of CSR themes, relationship cultivation strategies, and multimodal types. To address RQs 1b, 2b, 2c, and 3b, the study examined the associations between CSR topics, relationship cultivation strategies, and multimodal types with social media user engagement through NB2. NB2 was selected for its minimal reliance on stringent statistical assumptions and its frequent application in communication studies. The results from the NB2 analysis highlighted effective associations, enabling a deeper exploration of the sub-dimensions within each effective relationship cultivation strategy. For example, if the Openness strategy was found to positively influence public engagement, further analysis would identify which sub-dimensions of Openness—such as history, description, or mission statements—were most influential in driving this engagement.

Sina Weibo was chosen as the study’s platform due to its popularity and its status as China’s largest social media platform. It provides a dynamic space where the public can express opinions, and corporations can disseminate information effectively [15].

### 4.3. Data collection

A Weibo crawler program in Python 3.4 was employed to retrieve publicly available content from Weibo, targeting the top ten automotive companies based on CAAM’s 2023 sales rankings (see Table 1). The dataset analyzed comprised publicly available posts from verified automobile company accounts on Sina Weibo. Data collection and analysis methods complied fully with Sina Weibo’s Terms of Service, and no private or personal information was collected. The crawler automatically collected posts from the official Weibo accounts of these companies for the entire year from January 1 to December 31, 2023. This one-year timeframe aligns with a common planning cycle in media research [52].

**Table 1:** List of Sampled Companies.

Ranking	Company Name	Official Weibo Page	Number of CSR Posts
1	VW	https://weibo.com/vgc2011	34
2	TOYOTA	https://weibo.com/u/2286294114	62
3	BYD	https://weibo.com/bydauto	61
4	HONDA	https://weibo.com/hondamotorchina	43
5	SGMW	https://weibo.com/u/3634148760	28
6	BENZ	https://weibo.com/mymb	26
7	NISSAN	https://weibo.com/chinanissan	42
8	BUICK	https://weibo.com/sgmbuick	28
9	HAVEL	https://weibo.com/u/1888373567	35
10	TESLA	https://weibo.com/teslaofficial	62

Note. The ranking was obtained from CAAM.

Initially, 1,147 Weibo posts were retrieved. These were then filtered to identify CSR-related content using keywords such as carbon emission, environment, pollution, donation, sales volumes, and ethical crises, resulting in 494 posts. After manually reviewing these to remove any irrelevant posts, such as advertisements or those lacking CSR content, the final sample consisted of 421 relevant posts. These posts collectively accumulated 1,011,135 likes, 84,983 shares, and 143,528 comments.

### 4.4. Coding

#### 4.4.1. Coding scheme.

**Fig 1:**
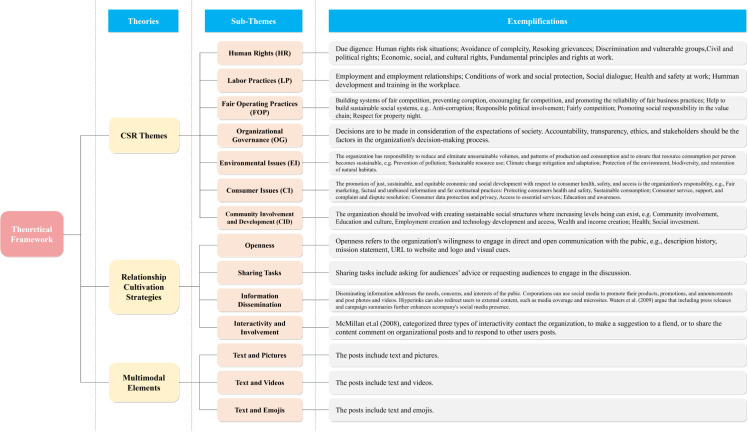
Proposed Theoretical Framework. *Note.* CSR themes are based on the ISO official website [49]. Relational strategies are drawn from Waters et al. [27], Ledingham and Bruning [50], and Kent and Taylor [31]. Multimodal types are influenced by Kress and van Leeuwen [47] and Jewitt et al. [48].

We developed an integrated coding scheme to address the research questions, aligning with the theoretical framework. The scheme consisted of three components: CSR themes, relationship cultivation strategies, and multimodal types. For RQ 1, which investigates CSR themes (RQ 1a), topics were identified in 421 posts using the ISO 26000. These topics included Human Rights (HR), Labor Practices (LP), Fair Operating Practices (FOP), Organizational Governance (OG), The Environment (TE), Consumer Issues (CI), and Community Involvement and Development (CID) (see Fig 1).

The second component of the coding scheme focused on relationship cultivation strategies, with categories derived from prior research [27,30,53]. Following Waters et al.’s approach to coding online relationship cultivation strategies, we analyzed CSR messages for the presence of four strategies: Openness, Sharing Tasks, Information Dissemination, and Interactivity and Involvement [27,30]. For Openness, we evaluated whether CSR messages included descriptions of the organization, its history, mission statements, links to the organization’s website, or logos and visual cues to foster relationships with the online public [30]. Sharing Tasks was coded based on actions such as soliciting advice and making direct or indirect requests. For Information Dissemination, we examined whether messages contained visual content (e.g., photographs, videos, emoticons), announcements, news links, or campaign summaries. To assess Interactivity and Involvement, we combined coding schemes from previous literature [27,30,53], capturing the interactive features of social media. This coding included elements such as organizational contact information (e.g., email, QR codes, addresses, phone numbers), navigation links to external content, opportunities for commenting and sharing, action features like polls, games, and quizzes, and whether corporate communicators responded to user comments [30]. The first two parts of the coding scheme used each sentence within the posts as the coding unit.

The final component of the coding scheme addressed multimodal types, which included combinations of text with pictures, videos, and emoticons. Unlike the first two components, the coding unit for multimodal types was each post, as a post could only have one multimodal type. Since individual sentences in a post cannot independently represent a multimodal type, each post was treated as a single coding unit for this dimension.

#### 4.4.2. Inter-rater reliability.

To minimize uncertainty in the coding process, coders underwent repeated training based on the coding scheme, and all disagreements were thoroughly discussed and resolved. To test intercoder reliability, 63 CSR posts from automotive companies—approximately 15% of the total dataset (421 posts)—were randomly selected and coded by two independent coders. Given the transient nature of web-based content, which can be removed or altered quickly [54], the coders completed the coding independently on the same day and at the same time. Cohen’s kappa values were calculated to assess intercoder reliability, yielding 0.89 for CSR topics, 0.79 for relationship cultivation strategies, and 0.95 for multimodal types, indicating strong agreement between the coders. After this reliability test, the remaining messages were divided equally, and each coder proceeded to code their assigned portion individually.

#### 4.4.3. Coding procedure.

The dataset of 421 posts contained a total of 1042 sentences, all of which were manually coded by the coders. For descriptive data, including the frequencies of CSR themes, communication strategies, and multimodal types, each sentence was treated as a coding unit and analyzed using the absence-presence coding method. To answer the research questions related to CSR themes (RQ 1s), coders carefully read each sentence and categorized it based on the first part of the coding scheme, which addressed the seven CSR themes. Following this, associations between the identified CSR themes and public engagement metrics were evaluated.

Next, using the second part of the coding scheme, coders examined the sentences for the presence of four relationship cultivation strategies. The frequencies of these strategies (see Fig 1) were calculated, with individual sentences being assigned to multiple categories if they included more than one CSR theme or strategy. Subsequently, the associations between each relationship cultivation strategy and public engagement metrics—such as likes, shares, and comments—were analyzed. Additionally, sub-dimensions of these strategies were identified, and their corresponding impact on public engagement on Weibo was investigated.

Similarly, to address the research questions related to multimodal types (RQ 3s), each selected post was analyzed and classified into one of three multimodal categories (see Fig 1). Since each post contained only one type of multimodal element, the coding unit for this analysis was the entire post, rather than individual sentences. The proportions of each multimodal type were calculated using SPSS. Finally, the associations between each multimodal type and public engagement metrics were examined.

### 4.5. Data analysis

#### 4.5.1. Descriptive analysis.

To address the frequency and proportion-related questions in the three RQs, a descriptive analysis was conducted. This included examining the frequencies of each CSR theme, relationship cultivation strategy, multimodal type, and inter-rater reliability. For RQ 1a and 2a, sentences were coded as 0 (“No”) or 1 (“Yes”). Public engagement, following Ji et al. [55], was measured by counting likes, shares, and comments. For RQ 3a, multimodal types in the selected posts were similarly coded as absent or present. SPSS was then used to calculate frequencies and identify the most commonly used multimodal types by automotive companies.

#### 4.5.2. Statistical Analysis.

In the social media context, RQ 1b, 2b, 2c, and 3b examined whether and how companies’ CSR themes, relationship cultivation strategies, and multimodal types influenced public engagement. Since the dependent variables (likes, shares, and comments) were count-based, a count regression model was used to evaluate these associations.

To identify the appropriate regression model, we assessed whether the data met the equidispersion assumption of Poisson regression (mean equals variance). Descriptive statistics, including variance-to-mean ratios, were calculated for likes (2879.8), shares (1750.8), and comments (157.6), as shown in Table 2.

**Table 2:** Descriptive Statistics and Dispersion Check for Dependent Variables.

Dependent Variable	Mean	Variance	Variance-to-Mean Ratio	Dispersion Indication
Likes	1200.45	3456789.12	2879.8	Overdispersed
Shares	450.67	789012.34	1750.8	Overdispersed
Comments	78.34	12345.67	157.6	Overdispersed

Note: Variance-to-mean ratios significantly greater than 1 indicate substantial overdispersion, confirming the necessity of using NB2 rather than Poisson regression [56,57].

Variance-to-mean ratios significantly greater than 1 confirmed substantial overdispersion, making Poisson regression unsuitable. Instead, consistent with prior research [56], NB2 was adopted. NB2 regression accommodates overdispersion by incorporating an additional dispersion parameter, providing robust and accurate modeling for highly dispersed count data. It is widely used in communication and social media research for its flexibility compared to Poisson regression [6].

After confirming the suitability of NB2 regression based on dispersion checks, we analyzed the relationships between public engagement and the use of CSR themes, relationship cultivation strategies, and multimodal message types.

## 5. Results and discussion

### 5.1 Results

#### 5.1.1 Descriptive results.

All the descriptive data in this study, including the frequencies and percentages of each CSR theme (RQ 1a), each relational cultivation strategy (RQ 2a), the sub-types of relational strategies (RQ 2c), each multimodal type (RQ 3a), inter-rater reliability and inter-rater agreement were counted and then analysed via SPSS. The corresponding descriptive results are presented in Table 3.

**Table 3:** Descriptive Information about Companies’ Weibo Posts in 2021.

Variable/Measure	Frequency	Percentage	Inter-rater Reliability	Agreement
CSR Themes				
HR	12	1.2%	.75	1
LP	10	1.0%	.81	1
FOP	16	1.5%	.76	1
OG	46	4.4%	.86	1
TE	272	26.1%	.89	1
CI	122	11.7%	.79	1
CID	387	37.1%	.92	1
Relationship Cultivation Strategy				
Openness/Disclosure	**628**	6.2%		1
Description	82	7.8%	.74	1
History	33	3.2%	.82	1
Mission Statement	53	5.1%	.79	1
URL to the Web Site	112	1.8%	.84	1
Logo/Visual Cues	348	33.4%	.93	1
Sharing of Tasks	**217**	2.8%		1
Asking for Advice	104	10%	.72	1
Direct/Indirect Request	113	1.8%	.68	1
Information Dissemination	**1042**	100%		1
News Links	64	6.1%	.93	1
Photo Posted	777	74.6%	.95	1
Video Files	75	7.2%	1.00	1
Announcements And Press Releases	62	6.0%	.76	1
Campaign Summaries	75	7.2%	.81	1
Interactivity and Involvement	**909**	87.2%		1
Organizational Contacts	18	1.7%	1.00	1
Navigation	448	43.0%	.63	1
Commenting Opportunity	52	5.0%	.89	1
Sharing to One’s Own Page	72	6.9%	.82	1
Action Features for Online Participation	59	5.7%	.83	1
Response to User Posts	260	25.0%	.96	1
Multimodal Types				
Text and Picture	315	74.8%	1.00	1
Text and Video	82	19.5%	1.00	1
text-emoji	45	5.7%	.98	1
Engagement of Social Media Users				
Shares	1011135			
Likes	84983			
Comments	143528			

*Note.* Cohen’s kappa is undefined for this variable due to invariant values.

In Table 3, inter-rater reliability exceeded 70%, with 100% agreement between raters, ensuring confidence in the coding results and validating the robustness of subsequent analyses.

For RQ 1a, the analysis revealed that Community Involvement and Development (CID) was the most frequently posted CSR theme among the automotive companies on Weibo, accounting for 37.1% of posts. This was followed by Environmental Issues (TE, 26.1%) and Consumer Issues (CI, 11.7%). Less common themes included Organizational Governance (OG, 4.4%), Fair Operating Practices (FOP, 1.5%), Human Rights (HR, 1.2%), and Labor Practices (LP, 1.0%). These results indicate a strong focus on CID and TE themes in the companies’ CSR communication.

Regarding RQ 2a, Information Dissemination emerged as the most frequently used relationship cultivation strategy, present in all posts (n = 1042, 100%). Interactivity and Involvement followed (n = 909, 87.2%), along with Openness (n = 628, 60.2%) and Sharing Tasks (n = 217, 20.8%). These findings suggest that companies prioritize Information Dissemination as their primary strategy for engaging Weibo users, while other strategies play smaller roles.

For RQ 2c, photographs were the most commonly used sub-dimension within the Information Dissemination strategy, appearing in 74.6% of posts (n = 777). Other sub-dimensions were used far less frequently, including video files (7.2%), campaign summaries (7.2%), news links (6.1%), and announcements/press releases (6.0%). To demonstrate Openness, companies frequently used logos and visual cues (n = 348, 33.4%) or included URLs (n = 112, 10.8%) to direct users to additional resources. Fewer posts included the company’s mission (n = 53, 5.1%) or company history (n = 33, 3.2%), reflecting a reliance on visual and navigational tools to foster Openness.

The Interactivity and Involvement strategy was mainly implemented through the navigation sub-dimension (n = 448, 43.0%). Companies also responded to user posts (n = 260, 25.0%), yet opportunities for engagement, such as commenting (n = 52, 5.0%), sharing posts (n = 72, 6.9%), or participating in online actions (n = 59, 5.7%), were rarely provided. Additionally, posts seldom included organizational contact details (n = 18, 1.7%), indicating limited use of deeper interactive features.

For RQ 3a, multimodal elements appeared in nearly all CSR posts. The most common type was text and picture (74.8%), with photographs frequently used to enrich content and engage users. The text and video type appeared in 19.5% of posts (n = 82), while the text and emoticon type was rare, found only in Tesla’s posts (n = 45, 5.7%).

#### 5.1.2 Statistical results.

To address RQ 1b, 2b, and 3b, NB2 regression was then used to examine associations between these factors and the number of likes, shares, and comments. The results are presented in Table 4.

**Table 4:** Negative Binomial Results for the CSR Topics, Relationship Cultivation Strategies, Multimodal Types, and the Number of Shares, Likes and Comments.

Predictor Variables	Numbers of Likes						Numbers of Shares						Numbers of comments					
*B* *(Coef.)*	*SE*	*z*	*P > |z|*	*95% CI*		*B*	*SE*	*z*	*P > |z|*	*95% CI*		*B*	*SE*	*z*	*P > |z|*	*95% CI*	
				*LL*	*UL*					*LL*	*UL*					*LL*	*UL*
**CSR Themes**																		
HR	−1.44	.49	−2.94	.001***	−2.40	−.48	−2.94	.59	−4.96	.01***	−4.10	−1.78	−.57	.49	−1.16	.25	−1.54	.39
LP	.45	.54	.83	.41	−.61	1.52	−.29	.65	−.45	.65	−1.57	.98	−.14	.53	−.27	.79	−1.18	.89
FOP	.81	.65	1.24	.22	−.47	2.09	−.51	.62	−.82	.41	−1.73	.71	1.27	.43	2.96	.01***	.43	2.11
OG	.16	.27	.58	.56	−.38	.69	.79	.32	2.46	.01***	.16	1.42	−.45	.27	−1.66	.10	−.99	.08
TE	−.26	.12	−2.14	.03***	−.50	−.02	−.30	.15	−2.03	.04***	−.58	−.01	−.19	.10	−1.78	.08	−.39	.02
CI	.66	.08	8.60	.01***	.51	.81	.31	.10	3.19	.003***	.12	.50	.58	.07	8.17	.002***	.44	.72
CID	.12	.08	1.44	.15	−.04	.28	−.02	.10	−.23	.82	−.22	.17	.07	.08	.93	.35	−.08	.23
**Relationship Cultivation Strategy**																		
Openness/ Disclosure	−.07	.09	−.80	.42	−.25	.10	.002	.12	.01	.99	−.23	.228	.19	.095	1.98	.048***	.002	.38
Sharing of Tasks	−.30	.13	−2.29	.02***	−.55	−.04	.47	.17	2.75	.006***	.14	.810	−.07	.15	−.48	.63	−.36	.22
Information Dissemination	.12	.04	3.52	.001***	.06	.19	.007	.05	−.17	.87	−.09	.075	.09	.04	2.54	.011***	.02	.16
Interactivity and Involvement	.03	.05	.65	.04***	−.12	.06	.14	.05	2.87	.004***	.045	.241	.15	.050	3.00	.003***	.052	.25
**Multimodal Types**																		
Text and Picture	−2.40	.55	−4.38	.001***	−3.47	−1.33	−1.38	.51	−2.71	.007***	−2.39	−.38	−1.86	.56	−3.33	.001***	−2.95	−.76
Text and Video	−2.27	.58	−3.9	.001***	−3.41	−1.13	−1.071	.56	−1.92	.055	−2.17	.03	−1.79	.59	−3.01	.003***	−2.95	−.62
text-emoji	.27	.31	.47	.04***	−.34	.88	−1.74	.36	−4.77	.01***	−2.45	−1.03	1.28	.31	4.13	.01***	.67	1.89

*Note:* Bold font denotes the significant p-value at.05.

As shown in Table 4, the NB2 results indicated that only the CI theme was positively associated with all three public engagement metrics on Weibo: likes (*Coef./B* = .66, *p > /z/ = *.01), shares (*Coef./B* = .31, *p > /z/ = *.003) and comments (*Coef./B = .*58*, p > /z/ = *.002); which means that CI CSR themes were able to generate more shares, likes and comments. Similarly, the OG theme was associated positively with the number of shares (*Coef./B = .*79*, p > /z/ = *.01), while FOP were positively associated with comments.

However, negative associations were found for some themes. For instance, the HR theme was negatively associated with likes (*Coef./B = −*1.44 *p > /z/ = *.001) and shares (*Coef./B* = −2.94*, p > /z/ = *.01), indicating it reduces user engagement. Similarly, the TE theme was negatively associated with likes (*Coef./B* = −.26 *p > /z/ = *.03) and shares (*Coef./B* = −.30*, p > /z/ = *.04).

Relationship Cultivation strategies also showed mixed effects on public engagement. The Openness strategy was positively associated with comments (*Coef. = *.19*, p > /z/ = *.048), which indicated Openness could generate more comments from social media users. The Sharing Tasks strategy was positively related to the number of shares (*Coef. = .*47*, p > /z/ = .*006). Similarly, there was a positive association between the *Information* Dissemination strategy and users’ likes (*Coef. = *.12*, p > /z/ = *.001), as well as comments (*Coef. = *.09*, p > /z/ = *.011). For the Interactivity and Involvement strategy, it was positively associated with the number of shares (*Coef. = *.14*, p > /z/ = *.004) and comments (Coef. *= *.15*, p > /z/ = *.003).

Regarding multimodal types, both text and picture and text and video types were negatively associated with all three engagement metrics. For text and picture, coefficients were as follows: likes (*Coef.* for text and picture = −2.40 and *Coef.* for text and video = −2.27), shares (*Coef. = *−1.38 and −1.07, respectively) and comments (*Coef. *= −1.86 and −1.79, respectively). The results for the *p*-value further revealed that the text and picture type could reduce all three dependent variables, as follows: the number of likes (*p > /z/ = *.001), shares (*p > /z/ = *.007) and comments (*p > /z/ = *.001). Similarly, the text and video multimodal types generated fewer likes (*p > /z/ = *.001) and comments (*p > /z/ = *.003). Finally, the results for the text and emoticon type differed from the other two multimodal types. With regard to shares and comments, the text and emoticon type was negatively associated with shares (*Coef. = *−1.739*, p > /z/ = *.01), but was associated positively with the number of comments (*Coef. = *1.281*, p > /z/ = *.01).

Table 4 revealed positive associations between the Sharing Tasks strategy and shares, the Openness strategy and comments, the Information Dissemination strategy and likes/comments, and the Interactivity and Involvement strategy and shares/comments. However, the specific sub-dimensions of these strategies influencing engagement remain unclear, requiring further exploration (See Table 5).

**Table 5:** The Results for the Sub-dimensions of Relationship Cultivation Strategies and Public Engagement.

Predictor variables	Numbers of likes						Numbers of shares						Numbers of comments					
*B*	*SE*	*z*	*P > |z|*	*95% CI*		*B*	*SE*	*z*	*P > |z|*	*95% CI*		*B*	*SE*	*z*	*P > |z|*	*95% CI*	
				*LL*	*UL*					*LL*	*UL*					*LL*	*UL*
**Openness/Disclosure**																		
Description	1.06	.23	4.68	.001	.62	1.51	1.20	.29	4.20	.001	.64	1.76	1.00	.25	4.00	.001**	.51	1.48
History	.02	.27	.08	.94	−.51	.55	.70	.49	1.42	.16	−.27	1.66	.04	.33	.12	.91	−.62	.69
Mission Statement	.08	.25	.33	.74	−.40	.56	.15	.33	.45	.65	−.50	.80	−.61	.29	−2.12	.03**	−1.17	−.05
URL to the Web Site	−.70	.16	−4.42	.001	−1.01	−.39	−.20	.26	−.76	.45	−.70	.31	−.99	.18	−5.62	.001**	−1.34	−.65
Logo/Visual Cues	−1.48	.22	−6.72	.001	−1.91	−1.05	−.65	.27	−2.38	.02	−1.19	−.12	−.66	.22	−2.97	.001**	−1.09	−.22
**Sharing of Tasks**																		
Asking for Advice	−.23	.15	−1.51	.13	−.53	.07	.11	.35	.32	.75	−.58	.80	−.29	.13	−2.18	.03	−.55	−.03
Direct/Indirect Request	−.10	.18	−.53	.59	−.44	.25	−.10	.24	−.42	.67	−.56	.36	.38	.18	2.15	.03	.03	.73
**Information Dissemination**																		
News Links	.04	.24	.16	.87	−.44	.52	−.20	.25	−.82	.41	−.68	.28	−.27	.20	−1.37	.17	−.65	.12
Photo Posted	.06	.03	1.85	.07	.001**	.11	−.03	.04	−.78	.43	−.11	.05	.04	.03	1.11	.27	−.03	.10
Video Files	.30	.22	1.34	.18	−.14	.73	1.08	.34	3.19	.00	.42	1.75	−.20	.23	−.87	.39	−.65	.25
Announcements	1.45	.22	6.70	.001**	1.03	1.88	.73	.25	2.96	.00	.25	1.22	1.56	.21	7.33	.001**	1.14	1.98
Campaign Summaries	−.67	.22	−3.05	.001**	−1.09	−.24	−.52	.28	−1.88	.06	−1.07	.02	−.55	.21	−2.52	.012**	−.98	−.12
**Interactivity and Involvement**																		
Organizational Contacts	−.51	.23	−2.20	.03	−.97	−.06	−.30	.27	−1.12	.26	−.82	.22	−.86	.26	−3.38	.001**	−1.36	−.36
Navigation	−.11	.06	−1.80	.07	−.23	.01	.10	.08	1.26	.21	−.06	.26	−.07	.06	−1.09	.28	−.19	.05
Commenting Opportunity	1.37	.28	4.99	.001**	.83	1.91	2.16	.41	5.25	.001**	1.35	2.96	.41	.26	1.56	.02**	−.11	.92
Sharing to One’s Own Page	.37	.23	1.61	.11	−.08	.81	−.60	.33	−1.83	.07	−1.25	.04	.80	.24	3.41	.001**	.34	1.26
Action Features for Online Participation	−.27	.22	−1.24	.21	−.70	.16	.52	.36	1.44	.15	−.19	1.22	.64	.23	2.78	.01**	.19	1.09
Response to Posts	.00	.08	.02	.99	−.15	.15	.12	.11	1.10	.27	−.09	.33	.14	.09	1.65	.10	−.03	.32

Note: Bold font denotes the significant p-value at.0

Table 5 details the associations between the sub-dimensions of relationship cultivation strategies and public engagement on Weibo. Based on Table 4, only positive associations were analyzed, including those between the sub-dimensions of the Openness and comments, Sharing Tasks and shares, Information Dissemination and likes/comments, and Interactivity and Involvement and shares/comments.

For the Openness, only the “description” sub-dimension was positively associated with comments (*Coef. = *1.00). The other three sub-dimensions of Openness, namely the “mission statement” (*Coef. = *−.61*, p > /z/ = *.03), the *“*URL to the website” (*Coef. = *−.99*, p > /z/ = *.001) and the “logo/visual cues” (*Coef. = *−.66*, p > /z/ = *.001) were all associated negatively with comments. With regard to the sub-dimensions of “Sharing Tasks,” the p-value of both sub-dimensions (“asking for advice and request”) were all over.05, which means that neither of the sub-dimensions could influence likes and shares.

For the Information Dissemination, posted “photographs” positively influenced likes (*Coef. = *.06, *p > /z/ = *.001). “Announcements” could generate more likes (*Coef. = *1.45, *p > /z/ = *.001) and comments (*Coef. = *1.56, *p > /z/ = *.001), whereas the campaign summaries could reduce the number of likes (*Coef. = *−.67*, p > /z/ = *.001).

For the Interactivity and Involvement, the “commenting opportunity” sub-dimension was positively associated with shares (*Coef. = *2.16*, p > /z/ *= .001). However, the organizational contacts sub-dimension was associated negatively with comments (*Coef. = *−.86*, p > /z/ = *0.001). From the result for organizational contacts, we found that organizational contacts elicited fewer comments on Weibo, but did not impact the number of shares. Similarly, sharing to one’s own page was associated positively with comments (*Coef. = *.80*, p > /z/ = *0.001). Action features for online participation also generated more comments (*Coef. = *.64*, p > /z/ = *.01).

To conclusion, the “description” sub-dimension of Openness increased comments, while photographs and announcements generated more likes and comments. “Commenting opportunities” encouraged more shares, while “sharing to one’s own page” and “online participation” features prompted higher comment engagement.

### 5.2. Discussion

This study examined the CSR themes, relationship cultivation strategies, and multimodal types used in automotive companies’ Weibo posts, as well as their influence on users’ online engagement. The following discussion highlights how these results compare to previous literature and offers actionable managerial recommendations based on the findings.

#### 5.2.1. Effects of CSR themes on users’ engagement.

According to the results, Consumer Issues (CI) topics generated more likes, shares, and comments on Weibo, even though CI-related messages were rarely posted by the automotive companies studied. This finding differs from Du and Vieira’s [58] study, which identified the environmentally related theme as the most frequent in CSR messages. High engagement with CI themes was particularly evident in posts addressing consumers’ health and safety issues. For example, many social media users interacted with posts from Honda or Tesla that discussed car quality, recalls, or accidents.

These findings extend previous research by emphasizing the importance of CI-themed CSR communication that directly affect users’ engagement. From the perspective of S-R theory, CI posts act as stimuli that resonate strongly with users’ personal safety concerns, prompting an immediate and engaged response. Moreover, U&G theory explained that users actively seek and engage with content that fulfills their need for safety and relevant information. Thus, managers should prioritize communicating CI-themed CSR topics—especially those related to health, safety, and product quality—to effectively capture audience attention and enhance engagement.

The results also revealed that the Community Involvement and Development (CID) theme was the most frequently posted CSR theme. This finding aligns with Ngai and Singh’s [6] study, which found CID themes to be the most common in CSR communications. This reflects corporations’ recognition of the public importance of CID issues [59]. However, our results showed that the association between CID themes and public engagement was weak. This contrasts with prior studies that emphasized CID’s importance to audiences [6]. One possible explanation is that CID issues encompass a wide range of topics, and engagement may vary depending on how closely specific issues resonate with audiences. For example, Smith and Alcorn [60] found that while half of consumers valued corporate support for charitable organizations, many did not actively engage or connect with philanthropic CSR initiatives. Similarly, Polonsky and Speed [61] argued that broad philanthropic efforts often lack clear consumer benefits, limiting public involvement.From an S-R perspective, CID themes may fail to serve as strong stimuli to drive engagement. This can also be explained through the lens of U&G theory, as users may not find the topic personally relevant or aligned with their interests, resulting in lower engagement. Managers should therefore carefully evaluate and select CID-related initiatives that match audience values, rather than broadly investing in philanthropic programs with limited engagement potential.

The results for the Environmental (TE) theme also contradicted prior findings. Studies such as Follows and Jobber [62] and Lin et al. [63] emphasized the value of environmental messages in shaping public perceptions and fostering brand loyalty. However, our findings showed that TE topics did not generate significant engagement on Weibo. A likely explanation is that the technical language used in TE posts acts as a barrier to public interaction. Posts often included terminology such as “carbon neutrality,” “emissions reduction,” and “environmental turbocharging,” which may confuse or alienate users. According to U&G theory, audiences prefer content that is easy to understand and fulfills their informational needs [45]. When TE messages are too complex, users may struggle to find relevance or utility, reducing their motivation to engage. To address this, managers should simplify technical language and contextualize environmental efforts in relatable terms, making CSR messages more accessible and engaging to a wider audience.

The findings highlight the importance of tailoring CSR communication strategies to audience needs and preferences. CI themes effectively engage users by addressing their safety concerns, while CID initiatives must be carefully selected to resonate with target audiences. Environmental messages should be simplified to maximize their appeal and accessibility.

#### 5.2.2. Effects of relationship cultivation strategies on user’s engagement.

The results demonstrated that Relationship strategies remain important in Weibo posts, though their effectiveness varies across dependent variables (likes, shares, comments). Among the four strategies, Openness, Information Dissemination, and Interactivity and Involvement positively influenced at least one engagement metric. Both Sharing Tasks and Interactivity strategies were effective in generating shares. This finding partially aligns with Sun et al.’s [64] research, which showed that Sharing Tasks could predict shares. Kim and Yang [23] also suggested that Sharing Tasks stimulate sharing behavior by combining rational information with visual content. However, our findings diverged from previous studies that suggested Sharing Tasks, such as “asking for audience advice,” were more likely to generate comments on platforms like Twitter [64]. These differences may stem from the cultural and contextual differences between Western platforms and Chinese social media, as most prior studies focused on Western contexts [28]. From S-R perspective, Sharing Tasks posts can be understood as stimuli that trigger user reactions, such as sharing, due to their clear call-to-action elements. Managers should therefore design posts that explicitly invite user participation and sharing, tailoring strategies to the preferences of audiences in the Chinese social media context, where sharing behaviors may be influenced by different cultural norms.

The Interactivity strategy was positively associated with shares and comments, consistent with prior findings that interactivity promotes two-way communication and is a key driver of public engagement on social media [32]. automotive companies often employed this strategy by incorporating navigation features, responding to comments, and adding action-oriented features for online participation. Posts with the sub-dimension “commenting opportunity” were effective, eliciting more likes, shares, and comments. For instance, posts asking users to share their preferred car models received high engagement. This supports prior research showing that messages soliciting user responses and providing rational, relatable content are strong predictors of engagement [23]. From a U&G perspective, posts inviting comments fulfill users’ needs for social interaction and self-expression, motivating them to engage actively [46]. By offering opportunities for personalized response, these posts align with users’ desire for meaningful participation. Managers should therefore incorporate direct questions and interactive elements into their posts to encourage user involvement, such as polls, open-ended questions, or invitations to share opinions.

#### 5.2.3. Effects of multimodal types on users’ engagement.

In general, posts with multimodal elements engaged more users. Public engagement increased when users read text-and-emoticon posts. However, results differed from previous predictions regarding text-picture and text-video types. Prior studies indicated that videos promote public engagement [23,24], but our results indicated the opposite.This may explained from U&G perspective that most social media users felt bored with frequently encountered text-picture combinations and avoided videos, perceiving them as a waste of time, thus lowering engagement. Managers should strategically use emoticons to enhance user engagement, and carefully evaluate video usage by producing concise, engaging content that respects audience time constraints and captures viewer interest quickly.

Overall, our findings partially align with previous studies but reveal important divergences. Like prior research [6], CID themes were commonly used in corporate CSR communications; however, contrary to expectations [6,59], they did not significantly enhance user engagement. Similarly, while environmental CSR topics are often considered effective for public engagement [62,63], our findings showed limited impact, likely due to overly technical language. Additionally, our results diverged from prior studies [23,24] by showing lower engagement for text-video posts. These differences highlight the role of contextual factors, such as cultural variations and message complexity, which future research should investigate further.

#### 5.2.4. Implications.

This study offers both theoretical and practical contributions to the field of CSR communication on social media. Theoretically, it integrates S-R theory and U&G theory to analyze CSR communication effectiveness. By combining S-R’s focus on external stimuli with U&G’s emphasis on users’ active motivations, this framework provides a comprehensive understanding of user engagement. For instance, CSR posts featuring CI themes serve as strong stimuli while also fulfilling users’ informational and emotional needs (U&G), resulting in higher engagement. This integrative approach deepens the theoretical understanding of how different types of CSR content provoke public reactions and provide a reference for future research in digital communication.

Practically, the findings emphasize the importance of tailoring CSR strategies to user preferences. Managers should focus on CI topics, simplify technical language in environmental posts, and use interactive strategies like direct questions or calls-to-action to boost engagement. By aligning content with user needs and applying insights from S-R and U&G theories, companies can design more effective CSR posts and strengthen relationships with their social media audiences.

#### 5.2.5. Limitations and future research.

This study has several limitations that future research should address. First, the small sample size—ten companies and 421 CSR Weibo messages—along with the focus on shares, comments, and likes, limits the generalizability of the findings. Additionally, content analysis cannot establish causality among variables, nor does this study explore how public engagement translates to offline behaviors.

Future research could address these gaps by using diverse methodologies, such as experiments, interviews, surveys, or real-time online data analysis, to examine the impact of relational strategies on public engagement from both corporate and public perspectives. Furthermore, this study focused solely on Weibo within the Chinese cultural context. Future studies should include cross-cultural comparisons to explore how different cultural contexts shape social media communication strategies, enabling the development of culturally tailored approaches for effective engagement.
